# Social inequalities and emerging infectious diseases.

**DOI:** 10.3201/eid0204.960402

**Published:** 1996

**Authors:** P Farmer

**Affiliations:** Harvard Medical School, Department of Social Medicine, Boston, MA 02115, USA. pefarmer@bics.bwh.harvard.edu

## Abstract

Although many who study emerging infections subscribe to social-production-of-disease theories, few have examined the contribution of social inequalities to disease emergence. Yet such inequalities have powerfully sculpted not only the distribution of infectious diseases, but also the course of disease in those affected. Outbreaks of Ebola, AIDS, and tuberculosis suggest that models of disease emergence need to be dynamic, systemic, and critical. Such models--which strive to incorporate change and complexity, and are global yet alive to local variation--are critical of facile claims of causality, particularly those that scant the pathogenic roles of social inequalities. Critical perspectives on emerging infections ask how large-scale social forces influence unequally positioned individuals in increasingly interconnected populations; a critical epistemology of emerging infectious diseases asks what features of disease emergence are obscured by dominant analytic frameworks. Research questions stemming from such a reexamination of disease emergence would demand close collaboration between basic scientists, clinicians, and the social scientists and epidemiologists who adopt such perspectives.


### Social Inequalities and Emerging Infectious Diseases

**Paul Farmer**
Harvard Medical School and
Brigham and Women's Hospital
Boston, Massachusetts, USA

Although many who study emerging infections subscribe to social-production-of-disease theories, few have examined the contribution of social inequalities to disease emergence. Yet such inequalities have powerfully sculpted not only the distribution of infectious diseases, but also the course of disease in those affected. Outbreaks of Ebola, AIDS, and tuberculosis suggest that models of disease emergence need to be dynamic, systemic, and critical. Such models—which strive to incorporate change and complexity, and are global yet alive to local variation—are critical of facile claims of causality, particularly those that scant the pathogenic roles of social inequalities. Critical perspectives on emerging infections ask how large-scale social forces influence unequally positioned individuals in increasingly interconnected populations; a critical epistemology of emerging infectious diseases asks what features of disease emergence are obscured by dominant analytic frameworks. Research questions stemming from such a reexamination of disease emergence would demand close collaboration between basic scientists, clinicians, and the social scientists and epidemiologists who adopt such perspectives.

The past decade has been one of the most eventful in the long history of infectious diseases. There are multiple indexes of these events and of the rate at which our knowledge base has grown. The sheer number of relevant publications indicates explosive growth; moreover, new means of monitoring antimicrobial resistance patterns are being used along with the rapid sharing of information (as well as speculation and misinformation) through means that did not exist even 10 years ago. Then there are the microbes themselves. One of the explosions in question—perhaps the most remarked upon—is that of "emerging infectious diseases." Among the diseases considered "emerging," some are regarded as genuinely new; AIDS and Brazilian purpuric fever are examples. Others have newly identified etiologic agents or have again burst dramatically onto the scene. For example, the syndromes caused by Hantaan virus have been known in Asia for centuries but now seem to be spreading beyond Asia because of ecologic and economic transformations that increase contact between humans and rodents. Neuroborreliosis was studied long before the monikers Lyme disease and *Borrelia burgdorferi* were coined, and before suburban reforestation and golf courses complicated the equation by creating an environment agreeable to both ticks and affluent humans. Hemorrhagic fevers, including Ebola, were described long ago, and their etiologic agents were in many cases identified in previous decades. Still other diseases grouped under the "emerging" rubric are ancient and well-known foes that have somehow changed, in pathogenicity or distribution. Multidrug-resistant tuberculosis (TB) and invasive or necrotizing Group A streptococcal infection are cases in point.

Like all new categories, "emerging infectious diseases" has benefits and limitations. The former are well known: a sense of urgency, notoriously difficult to arouse in large bureaucracies, has been marshaled, funds have been channeled, conferences convened, articles written, and a journal dedicated to the study of these diseases has

Address for correspondence: Paul Farmer, Harvard Medical School, Department of Social Medicine, 641 Huntington Avenue, Boston, MA 02115 USA; fax: 617-432-2565; e-mail: pefarmer@bics.bwh.harvard.edu.

been founded. The research and action agendas elaborated in response to the perceived emergence of new infections have been, by and large, sound. But the concept, like some of the diseases associated with it, is complex. Its complexity has, in some instances, hampered the learning process. A richly textured understanding of emerging infections will be grounded in critical and reflexive study of how learning occurs. Units of analysis and key terms will be scrutinized and defined more than once. This process will include regular rethinking not only of methods and study design, but also of the validity of causal inference and reflection on the limits of human knowledge. This study of the process, loosely known as epistemology, often happens in retrospect, but many of the chief contributors to the growing research in emerging infectious diseases have examined the epistemologic issues surrounding their work and are familiar with the multifactorial nature of disease emergence: "Responsible factors include ecological changes, such as those due to agricultural or economic development or to anomalies in the climate; human demographic changes and behavior; travel and commerce; technology and industry; microbial adaptation and change; and breakdown of public health measures" (1). A recent Institute of Medicine report on emerging infections does not even categorize microbial threats by type of agent, but rather according to factors held to be related to their emergence (2).

In studying emerging infectious diseases, many thus make a distinction between a host of phenomena directly related to human actions—from improved laboratory techniques and scientific discovery to economic "development," global warming, and failures of public health—and another set of phenomena, much less common and related to changes in the microbes themselves. Close examination of microbial mutations often shows that, again, human actions have played a large role in enhancing pathogenicity or increasing resistance to antimicrobial agents. In one long list of emerging viral infections, for example, only the emergence of Rift Valley fever is attributed to a possible change in virulence or pathogenicity, and this only after other, social factors for which there is better evidence (1). No need, then, to call for a heightened awareness of the sociogenesis, or "anthropogenesis," of emerging infections. Some bench scientists in the field are more likely to refer to social factors and less likely to make immodest claims of causality about them than are behavioral scientists who study disease. Yet a critical epistemology of emerging infectious diseases is still in its early stages of development; a key task of such a critical approach would be to take existing conceptual frameworks, including that of disease emergence, and ask, What is obscured in this way of conceptualizing disease? What is brought into relief? A first step in understanding the "epistemological dimension" of disease emergence, notes Eckardt, involves developing "a certain sensitivity to the terms we are used to" (3).

A heightened sensitivity to other common rubrics and terms shows that certain aspects of disease emergence are brought into relief while others are obscured. When we think of "tropical diseases," malaria comes quickly to mind. But not too long ago, malaria was an important problem in areas far from the tropics. Although there is imperfect overlap between malaria as currently defined and the malaria of the mid-19th century, some U.S. medical historians agree with contemporary assessments: malaria "was the most important disease in the country at the time." In the Ohio River Valley, according to Daniel Drake's 1850 study, thousands died in seasonal epidemics. During the second decade of the 20th century, when the population of 12 southern states was approximately 25 million, an estimated million cases of malaria occurred each year. Malaria's decline in this country was "due only in small part to measures aimed directly against it, but more to agricultural development and other factors some of which are still not clear" (4). These factors include poverty and social inequalities, which led, increasingly, to differential morbidity with the development of improved housing, land drainage, mosquito repellents, nets, and electric fans—all well beyond the reach of those most at risk for malaria. In fact, many "tropical" diseases predominantly affect the poor; the groups at risk for these diseases are often bounded more by socioeconomic status than by latitude.

Similarly, the concept of "health transitions" is influential in what some have termed "the new public health" and in the international financial institutions that so often direct development efforts (5). The model of health transitions suggests that nation-states, as they develop, go through predictable epidemiologic transformations. Death due to infectious causes is supplanted by death due to malignancies and to complications of coronary artery disease, which occur at a more advanced age, reflecting progress. Although it describes broad patterns now found throughout the world, the concept of national health transitions also masks other realities, including intranational illness and death differentials that are more tightly linked to local inequalities than to nationality. For example, how do the variables of class and race fit into such paradigms? In Harlem, where the age-specific death rate in several groups is higher than in Bangladesh, leading causes of death are infectious diseases and violence (6).

Units of analysis are similarly up for grabs. When David Satcher, director of the Centers for Disease Control and Prevention (CDC), writing of emerging infectious diseases, reminds us that "the health of the individual is best ensured by maintaining or improving the health of the entire community" (7), we should applaud his clearsightedness but go on to ask, What constitutes "the entire community"? In the 1994 outbreak of cryptosporidiosis in Milwaukee, for example, the answer might be "part of a city" (8). In other instances, community means a village or the passengers on an airplane. But the most common unit of analysis in public health, the nation-state, is not all that relevant to organisms such as dengue virus, *Vibrio cholerae* O139, human immunodeficiency virus (HIV), penicillinase-producing *Neisseria gonorrhoeae*, and hepatitis B virus. Such organisms have often ignored political boundaries, even though their presence may cause a certain degree of turbulence at national borders. The dynamics of emerging infections will not be captured in national analyses any more than the diseases are contained by national boundaries, which are themselves emerging entities—most of the world's nations are, after all, 20th-century creations.

Here I have discussed the limitations of three important ways of viewing the health of populations—tropical medicine, "the" epidemiologic transition, and national health profiles—because models and even assumptions about infectious diseases need to be dynamic, systemic, and critical. That is, models with explanatory power must be able to track rapidly changing clinical, even molecular, phenomena and link them to the large-scale (sometimes transnational) social forces that manifestly shape the contours of disease emergence. I refer, here, to questions less on the order of how pig-duck agriculture might be related to the antigenic shifts central to influenza pandemics, and more on the order of the following: Are World Bank policies related to the spread of HIV, as has recently been claimed (9)? What is the relationship between international shipping practices and the spread of cholera from Asia to South America and elsewhere in the Western Hemisphere (10,11)? How is genocide in Rwanda related to cholera in Zaire (12)?

The study of anything said to be emerging tends to be dynamic. But the very notion of emergence in heterogeneous populations poses questions of analysis that are rarely tackled, even in modern epidemiology, which, as McMichael has recently noted, "assigns a primary importance to studying interindividual variations in risk. By concentrating on these specific and presumed free-range individual behaviors, we thereby pay less attention to the underlying social-historical influences on behavioral choices, patterns, and population health" (13). A critical (and self-critical) approach would ask how existing frameworks might limit our ability to discern trends that can be linked to the emergence of diseases. Not all social-production-of-disease theories are equally alive to the importance of how relative social and economic positioning—inequality—affects risk for infection. In its report on emerging infections, the Institute of Medicine lists neither poverty nor inequality as "causes of emergence" (2).

A critical approach pushes the limits of existing academic politesse to ask harder and rarely raised questions: What are the mechanisms by which changes in agriculture have led to outbreaks of Argentine and Bolivian hemorrhagic fever, and how might these mechanisms be related to international trade agreements, such as the General Agreement on Tariffs and Trade and the North American Free Trade Agreement? How might institutional racism be related to urban crime and the outbreaks of multidrug-resistant TB in New York prisons? Does the privatization of health services buttress social inequalities, increasing risk for certain infections—and death—among the poor of sub-Saharan Africa and Latin America? How do the colonial histories of Belgium and Germany and the neocolonial histories of France and the United States tie in to genocide and a subsequent epidemic of cholera among Rwandan refugees? Similar questions may be productively posed in regard to many diseases now held to be emerging.

### Emerging How and to What Extent? The Case of Ebola

Hemorrhagic fevers have been known in Africa since well before the continent was dubbed "the white man's grave," an expression that, when deployed in reference to a region with high rates of premature death, speaks volumes about the differential valuation of human lives. Ebola itself was isolated fully two decades ago (14). Its appearance in human hosts has at times been insidious but more often takes the form of explosive eruptions. In accounting for recent outbreaks, it is unnecessary to postulate a change in filovirus virulence through mutation. The Institute of Medicine lists a single "factor facilitating emergence" for filoviruses: "virus-infected monkeys shipped from developing countries via air" (2).

Other factors are easily identified. Like that of many infectious diseases, the distribution of Ebola outbreaks is tied to regional trade networks and other evolving social systems. And, like those of most infectious diseases, Ebola explosions affect, researchers aside, certain groups (people living in poverty, health care workers who serve the poor) but not others in close physical proximity. Take, for example, the 1976 outbreak in Zaire, which affected 318 persons. Although respiratory spread was speculated, it has not been conclusively demonstrated as a cause of human cases. Most expert observers thought that the cases could be traced to failure to follow contact precautions, as well as to improper sterilization of syringes and other paraphernalia, measures that in fact, once taken, terminated the outbreak (15). On closer scrutiny, such an explanation suggests that Ebola does not emerge randomly: in Mobutu's Zaire, one's likelihood of coming into contact with unsterile syringes is inversely proportional to one's social status. Local élites and sectors of the expatriate community with access to high-quality biomedical services (viz., the European and American communities and not the Rwandan refugees) are unlikely to contract such a disease.

The changes involved in the disease's visibility are equally embedded in social context. The emergence of Ebola has also been a question of our consciousness. Modern communications, including print and broadcast media, have been crucial in the construction of Ebola—a minor player, statistically speaking, in Zaire's long list of fatal infections—as an emerging infectious disease (16). Through Cable News Network (CNN) and other television stations, Kikwit became, however briefly, a household word in parts of Europe and North America. Journalists and novelists wrote best-selling books about small but horrific plagues, which in turn became profitable cinema. Thus, symbolically and proverbially, Ebola spread like wildfire—as a danger potentially without limit. It emerged.

### Emerging From Where? The Case of TB

TB is said to be another emerging disease, in which case, emerging is synonymous with reemerging. Its recrudescence is often attributed to the advent of HIV—the Institute of Medicine lists "an increase in immunosuppressed populations" as the sole factor facilitating the resurgence of TB (2)—and the emergence of drug resistance. A recent book on TB, subtitled "How the battle against tuberculosis was won—and lost," argues that "Throughout the developed world, with the successful application of triple therapy and the enthusiastic promotion of prevention, the death rate from tuberculosis came tumbling down" (17). But was this claim ever documented? Granted, the discovery of effective anti-TB therapies has saved the lives of hundreds of thousands of TB patients, many in industrialized countries. But TB—once the leading cause of death among young adults in the industrialized world—was already declining there well before the 1943 discovery of streptomycin. In the rest of the world, and in pockets of the United States, TB remains undaunted by ostensibly effective drugs, which are used too late, inappropriately, or not at all: "It is sufficiently shameful," notes one of the world's leading authorities on TB, "that 30 years after recognition of the capacity of triple-therapy . . . to elicit 95%+ cure rates, tuberculosis prevalence rates for *many* nations remain unchanged" (18). Some estimate that more than 1.7 billion persons are infected with quiescent, but viable, *Mycobacterium tuberculosis* and, dramatic shifts in local epidemiology aside, a global analysis does not suggest major decreases in the importance of TB as a cause of death. TB has retreated in certain populations, maintained a steady state in others, and surged forth in still others, remaining, at this writing, the world's leading infectious cause of adult deaths (19).

At mid-century, TB was still acknowledged as the "great white plague." What explains the invisibility of this killer by the 1970s and 1980s? Again, one must turn to the study of disease awareness, that is, of consciousness and publicity, and their relation to power and wealth. "The neglect of tuberculosis as a major public health priority over the past two decades is simply extraordinary," wrote Murray in 1991. "Perhaps the most important contributor to this state of ignorance was the greatly reduced clinical and epidemiologic importance of tuberculosis in the wealthy nations" (20). Thus TB has not really emerged so much as emerged from the ranks of the poor (21,22). An implication, clearly, is that one place for diseases to hide is among poor people, especially when the poor are socially and medically segregated from those whose deaths might be considered more important.

When complex forces move more poor people into the United States, an increase in TB incidence is inevitable. In a recent study of the disease among foreign-born persons in the United States, immigration is essentially credited with the increased incidence of TB-related disease (23). The authors note that in some of the immigrants' countries of origin the annual rate of infection is up to 200 times that registered in the United States; moreover, many persons with TB in the United States live in homeless shelters, correctional facilities, and camps for migrant workers. But there is no discussion of poverty or inequality, even though these are, along with war, leading reasons for both the high rates of TB and for immigration to the United States. "The major determinants of risk in the foreign-born population," conclude the authors, "were the region of the world from which the person emigrated and the number of years in the United States."

### Going Where? The Case of HIV

To understand the complexity of the issues—medical, social, and communicational—that surround the emergence of a disease into public view, consider AIDS. In the early 1980s, the public was informed by health officials that AIDS had probably emerged from Haiti. In December 1982, for example, a physician affiliated with the National Cancer Institute was widely quoted in the popular press stating that "We suspect that this may be an epidemic Haitian virus that was brought back to the homosexual population in the United States" (24). This proved incorrect, but not before damage to Haitian tourism had been done. Result: more poverty, a yet steeper slope of inequality and vulnerability to disease, including AIDS. The label "AIDS vector" was also damaging to the million or so Haitians living elsewhere in the Americas and certainly hampered public health efforts among them (25).

HIV disease has since become the most extensively studied infection in human history. But some questions are much better studied than are others. And error is worth studying, too. Careful investigation of the mechanisms by which immodest claims are propagated (as regards Haiti and AIDS, these mechanisms included "exoticization" of Haiti, racism, the existence of influential folk models about Haitians and Africans, and the conflation of poverty and cultural difference) is an important yet neglected part of a critical epistemology of emerging infectious diseases. Also underinvestigated are considerations of the pandemic's dynamic. HIV may not have come from Haiti, but it was going to Haiti. Critical reexamination of the Caribbean AIDS pandemic showed that the distribution of HIV does not follow national borders, but rather the contours of a transnational socioeconomic order. Furthermore, much of the spread of HIV in the 1970s and 1980s moved along international "fault lines," tracking along steep gradients of inequality, which are also paths of migrant labor and sexual commerce (26).

In an important overview of the pandemic's first decade, Mann and coworkers observe that its course "within and through global society is not being affected—in any serious manner—by the actions taken at the national or international level" (27). HIV has emerged but is going where? Why? And how fast? The Institute of Medicine lists several factors facilitating the emergence of HIV: "urbanization; changes in lifestyles/mores; increased intravenous drug abuse; international travel; medical technology" (2). Much more could be said. HIV has spread across the globe, often wildly, but rarely randomly. Like TB, HIV infection is entrenching itself in the ranks of the poor or otherwise disempowered. Take, as an example, the rapid increase in AIDS incidence among women. In a 1992 report, the United Nations observed that "for most women, the major risk factor for HIV infection is being married. Each day a further three thousand women become infected, and five hundred infected women die" (28). It is not marriage per se, however, that places young women at risk. Throughout the world, most women with HIV infection, married or not, are living in poverty. The means by which confluent social forces, such as gender inequality and poverty, come to be embodied as risk for infection with this emerging pathogen have been neglected in biomedical, epidemiologic, and even social science studies on AIDS. As recently as October 1994—15 years into an ever-emerging pandemic—a *Lancet* editorial could comment, "We are not aware of other investigators who have considered the influence of socioeconomic status on mortality in HIV-infected individuals" (29). Thus, in AIDS, the general rule that the effects of certain types of social forces on health are unlikely to be studied applies in spite of widespread impressions to the contrary.

AIDS has always been a strikingly patterned pandemic. Regardless of the message of public health slogans—"AIDS is for Everyone"—some are at high risk for HIV infection, while others, clearly, are at lower risk. Furthermore, although AIDS eventually causes death in almost all HIV-infected patients, the course of HIV disease varies. Disparities in the course of the disease have sparked the search for hundreds of cofactors, from *Mycoplasma* and ulcerating genital lesions to voodoo rites and psychological predisposition. However, not a single association has been compellingly shown to explain disparities in distribution or outcome of HIV disease. The only well-demonstrated cofactors are social inequalities, which have structured not only the contours of the AIDS pandemic, but also the course of the disease once a patient is infected (30-33). The advent of more effective antiviral agents promises to heighten those disparities even further: a three-drug regimen that includes a protease inhibitor will cost $12,000 to $16,000 a year (34).

### Questions for a Critical Epistemology of Emerging Infectious Diseases

Ebola, TB, and HIV infection are in no way unique in demanding contextualization through social science approaches. These approaches include the grounding of case histories and local epidemics in the larger biosocial systems in which they take shape and demand exploration of social inequalities. Why, for example, were there 10,000 cases of diphtheria in Russia from 1990 to 1993? It is easy enough to argue that the excess cases were due to a failure to vaccinate (35). But only in linking this distal (and, in sum, technical) cause to the much more complex socioeconomic transformations altering the region's illness and death patterns will compelling explanations emerge (36,37).

Standard epidemiology, narrowly focused on individual risk and short on critical theory, will not reveal these deep socioeconomic transformations, nor will it connect them to disease emergence. "Modern epidemiology," observes one of its leading contributors, is "oriented to explaining and quantifying the bobbing of corks on the surface waters, while largely disregarding the stronger undercurrents that determine where, on average, the cluster of corks ends up along the shoreline of risk" (13). Neither will standard journalistic approaches add much: "Amidst a flood of information," notes the chief journalistic chronicler of disease emergence, "analysis and context are evaporating . . . Outbreaks of flesh eating bacteria may command headlines, but local failures to fully vaccinate preschool children garner little attention unless there is an epidemic" (38).

Research questions identified by various blue-ribbon panels are important for the understanding and eventual control of emerging infectious diseases (39,40). Yet both the diseases and popular and scientific commentary on them pose a series of corollary questions, which, in turn, demand research that is the exclusive province of neither social scientists nor bench scientists, clinicians, or epidemiologists. Indeed, genuinely transdisciplinary collaboration will be necessary to tackle the problems posed by emerging infectious diseases. As prolegomena, four areas of corollary research are easily identified. In each is heard the recurrent leitmotiv of inequality:

### Social Inequalities

Study of the reticulated links between social inequalities and emerging disease would not construe the poor simply as "sentinel chickens," but instead would ask, What are the precise mechanisms by which these diseases come to have their effects in some bodies but not in others? What propagative effects might social inequalities per se contribute (41)? Such queries were once major research questions for epidemiology and social medicine but have fallen out of favor, leaving a vacuum in which immodest claims of causality are easily staked. "To date," note Krieger and co-workers in a recent, magisterial review, "only a small fraction of epidemiological research in the United States has investigated the effects of racism on health" (42). They join others in noting a similar dearth of attention to the effects of sexism and class differences; studies that examine the conjoint influence of these social forces are virtually nonexistent (43,44).

And yet social inequalities have sculpted not only the distribution of emerging diseases, but also the course of disease in those affected by them, a fact that is often downplayed: "Although there are many similarities between our vulnerability to infectious diseases and that of our ancestors, there is one distinct difference: we have the benefit of extensive scientific knowledge" (7). True enough, but Who are "we"? Those most at risk for emerging infectious diseases generally do not, in fact, have the benefit of cutting-edge scientific knowledge. We live in a world where infections pass easily across borders—social and geographic—while resources, including cumulative scientific knowledge, are blocked at customs.

### Transnational Forces

"Travel is a potent force in disease emergence and spread," as Wilson has reminded us, and the "current volume, speed, and reach of travel are unprecedented" (45). Although the smallpox and measles epidemics following the European colonization of the Americas were early, deadly reminders of the need for systemic understandings of microbial traffic, there has been, in recent decades, a certain reification of the notion of the "catchment area." A useful means of delimiting a sphere of action—a district, a county, a country—is erroneously elevated to the status of explanatory principle whenever the geographic unit of analysis is other than that defined by the disease itself. Almost all diseases held to be emerging—from the increasing number of drug-resistant diseases to the great pandemics of HIV infection and cholera—stand as modern rebukes to the parochialism of this and other public health constructs (46). And yet a critical sociology of liminality—both the advancing, transnational edges of pandemics and also the impress of human-made administrative and political boundaries on disease emergence—has yet to be attempted.

The study of borders qua borders means, increasingly, the study of social inequalities. Many political borders serve as semipermeable membranes, often quite open to diseases and yet closed to the free movement of cures. Thus may inequalities of access be created or buttressed at borders, even when pathogens cannot be so contained. Research questions might include, for example, What effects might the interface between two very different types of health care systems have on the rate of advance of an emerging disease? What turbulence is introduced when the border in question is between a rich and a poor nation? Writing of health issues at the U.S.-Mexican border, Warner notes that "It is unlikely that any other binational border has such variety in health status, entitlements, and utilization" (47). Among the infectious diseases registered at this border are multidrug-resistant TB, rabies, dengue, and sexually transmitted diseases including HIV infection (said to be due, in part, to "cross-border use of 'red-light' districts").

Methods and theories relevant to the study of borders and emerging infections would come from disciplines ranging from the social sciences to molecular biology: mapping the emergence of diseases is now more feasible with the use of restriction fragment length polymorphism and other new technologies (48). Again, such investigations will pose difficult questions in a world where plasmids can move, but compassion is often grounded.

### The Dynamics of Change

Can we elaborate lists of the differentially weighted factors that promote or retard the emergence or reemergence of infectious diseases? It has been argued that such analyses will perforce be historically deep and geographically broad, and they will at the same time be processual, incorporating concepts of change. Above all, they will seek to incorporate complexity rather than to merely dissect it. As Levins has recently noted, "effective analysis of emerging diseases must recognize the study of complexity as perhaps the central general scientific problem of our time" (49). Can integrated mathematical modeling be linked to new ways of configuring systems, avoiding outmoded units of analyses, such as the nation-state, in favor of the more fluid biosocial networks through which most pathogens clearly move? Can our embrace of complexity also include social complexity and the unequal positioning of groups within larger populations? Such perspectives could be directed towards mapping the progress of diseases from cholera to AIDS, and would permit us to take up more unorthodox research subjects—for example, the effects of World Bank projects and policies on diseases from onchocerciasis to plague.

### Critical Epistemology

Many have already asked, What qualifies as an emerging infectious disease? More critical questions might include, Why do some persons constitute "risk groups," while others are "individuals at risk"? These are not merely nosologic questions; they are canonical ones. Why are some approaches and subjects considered appropriate for publication in influential journals, while others are dismissed out of hand? A critical epistemology would explore the boundaries of polite and impolite discussion in science. A trove of complex, affect-laden issues—attribution of blame to perceived vectors of infection, identification of scapegoats and victims, the role of stigma—are rarely discussed in academic medicine, although they are manifestly part and parcel of many epidemics.

Finally, why are some epidemics visible to those who fund research and services, while others are invisible? In its recent statements on TB and emerging infections, for example, the World Health Organization uses the threat of contagion to motivate wealthy nations to invest in disease surveillance and control out of self-interest—an age-old public health approach acknowledged in the Institute of Medicine's report on emerging infections: "Diseases that appear not to threaten the United States directly rarely elicit the political support necessary to maintain control efforts" (2). If related to a study under consideration, questions of power and control over funds, must be discussed. That they are not is more a marker of analytic failures than of editorial standards.

Ten years ago, the sociologist of science Bruno Latour reviewed hundreds of articles appearing in several Pasteur-era French scientific reviews to constitute what he called an "anthropology of the sciences" (he objected to the term epistemology). Latour cast his net widely. "There is no essential difference between the human and social sciences and the exact or natural sciences," he wrote, "because there is no more science than there is society. I have spoken of the Pasteurians as they spoke of their microbes" (50) (Here, perhaps, is another reason to engage in a "proactive" effort to explore themes usually relegated to the margins of scientific inquiry: those of us who describe the comings and goings of microbes—feints, parries, emergences, retreats—may one day be subjected to the scrutiny of future students of the subject).

Microbes remain the world's leading causes of death (51). In "The conquest of infectious diseases: who are we kidding?" the authors argue that "clinicians, microbiologists, and public health professionals must work together to prevent infectious diseases and to detect emerging diseases quickly" (52). But past experience with epidemics suggests that other voices and perspectives could productively complicate the discussion. In every major retrospective study of infectious disease outbreaks, the historical regard has shown us that what was not examined during an epidemic is often as important as what was (53,54) and that social inequalities were important in the contours of past disease emergence. The facts have taught us that our approach must be dynamic, systemic, and critical. In addition to historians, then, anthropologists and sociologists accountable to history and political economy have much to add, as do the critical epidemiologists mentioned above (55-58).

My intention, here, is ecumenical and complementary. A critical framework would not aspire to supplant the methods of the many disciplines, from virology to molecular epidemiology, which now concern themselves with emerging diseases. "The key task for medicine," argued the pioneers Eisenberg and Kleinman some 15 years ago, "is not to diminish the role of the biomedical sciences in the theory and practice of medicine but to supplement them with an equal application of the social sciences in order to provide both a more comprehensive understanding of disease and better care of the patient. The problem is not 'too much science,' but too narrow a view of the sciences relevant to medicine" (59).

A critical anthropology of emerging infections is young, but it is not embryonic. At any rate, much remains to be done and the tasks themselves are less clear perhaps than their inherent difficulties. The philosopher Michel Serres once observed that the border between the natural and the human sciences was not to be traced by clean, sharp lines. Instead, this border recalled the Northwest Passage: long and perilously complicated, its currents and inlets often leading nowhere, dotted with innumerable islands and occasional floes (60). Serres' metaphor reminds us that a sea change is occurring in the study of infectious disease even as it grows, responding, often, to new challenges—and sometimes to old challenges newly perceived.

### Acknowledgments

The author acknowledges the editorial suggestions of Cassis Henry, Harvard Medical School, and Haun Saussy, Stanford University.

*Dr. Farmer, an anthropologist/physician, is assistant professor of social medicine at the Harvard Medical School and divides his clinical practice between the Brigham and Women's Hospital and the Clinique Bon Sauveur in rural Haiti, where he directs the TB unit. His books*

*include* AIDS and Accusation *and* The Uses of Haiti*; he is the editor of* Women, Poverty and AIDS*:* Sex, Drugs, and Structural Violence.

### References

1. Morse S. Factors in the emergence of infectious diseases. Emerging Infectious Diseases 1995;1:7-15.
2. Lederberg J, Shope RE, Oaks SC. Emerging infections: microbial threats to health in the United States. Washington, D.C.: National Academy Press, 1992.
3. Eckardt I. Challenging complexity: conceptual issues in an approach to new disease. Ann New York Acad Sci 1994;740:408-17.
4. Levine N. Editor's preface to selections from Drake D. Malaria in the interior valley of North America. Urbana: University of Illinois Press, 1964 (1850).
5. Frenk J, Chacon F. Bases conceptuales de la nueva salud internacional. Salud Pública Méx 1991;33;307-13.
6. McCord C, Freeman H. Excess mortality in Harlem. N Engl J Med, 1990;322:173-7.
7. Satcher D. Emerging infections: getting ahead of the curve. Emerging Infectious Diseases 1995;1:1-6.
8. MacKenzie W, Hoxie N, Proctor M, Gradus MS, Blair KA, Peterson DE, et al. A massive outbreak in Milwaukee of Cryptosporidium infection transmitted through the water supply. N Engl J Med 1994;331:161-7.
9. Lurie P, Hintzen P, Lowe RA. Socioeconomic obstacles to HIV prevention and treatment in developing countries: the roles of the International Monetary Fund and the World Bank. AIDS 1995;9:539-46.
10. World Health Organization. Cholera in the Americas. Weekly Epidemiol Rec. 1992;67:33-9.
11. McCarthy S, McPhearson R, Guarino A. Toxigenic Vibrio cholera O1 and cargo ships entering the Gulf of Mexico. Lancet 1992;339:624.
12. Goma Epidemiology Group. Public health impact of Rwandan refugee crisis: what happened in Goma, Zaire, in July, 1994? Lancet 1995;345:339-44.
13. McMichael A. The health of persons, populations, and planets: epidemiology comes full circle. Epidemiology 1995;6:633-6.
14. Johnson KM, Webb PA, Lange JV, Murphy FA. Isolation and partial characterization of a new virus causing acute hemorrhagic fever in Zaire. Lancet 1977;1:569-71.
15. World Health Organization. Ebola haemorrhagic fever in Zaire, 1976. Report of an international commission. Bull WHO 1978;56:271-93.
16. Garrett L. The Coming Plague. New York: Farrar, Straus and Giroux, 1995.
17. Ryan F. The forgotten plague: how the battle against tuberculosis was won—and lost. Boston: Little, Brown, 1993.
18. Iseman M. Tailoring a time-bomb. Am Rev Respir Dis 1985;132:735-6.
19. Bloom B, Murray C. Tuberculosis: commentary on a resurgent killer. Science 1992;257:1055-63.
20. Murray C. Social, economic and operational research on tuberculosis: recent studies and some priority questions. Bull Int Union Tuberc Lung Dis 1991;66:149-56.
21. Farmer P, Robin S, Ramilus St-L, Kim J. Tuberculosis and "compliance": lessons from rural Haiti. Seminars in Respiratory Infections 1991;6:373-9.
22. Spence D, Hotchkiss J, Williams C, Davies P. Tuberculosis and poverty. British Medical Journal 1993;307:759-61.
23. McKenna MT, McCray E, Onorato I. The epidemiology of tuberculosis among foreign-born persons in the United States, 1986 to 1993. N Engl J Med 1995;332:1071-6.
24. Chabner B. Cited in the Miami News, December 2, 1982;8A.
25. Farmer P. AIDS and Accusation: Haiti and the Geography of Blame. University of California Press, Berkeley, 1992.
26. Farmer P. The exotic and the mundane: human immunodeficiency virus in the Caribbean. Human Nature 1990;1:415-45.
27. Mann J, Tarantola D, Netter T. AIDS in the world. Cambridge, MA: Harvard University Press, 1992.
28. United Nations Development Program. Young women: silence, susceptibility and the HIV epidemic. New York, UNDP, 1992.
29. Sampson J, Neaton J. On being poor with HIV. Lancet 1994;344:1100-1.
30. Chaisson RE, Keruly JC, Moore RD. Race, sex, drug use, and progression of human immunodeficiency virus disease. N Engl J Med 1995;333:751-6.
31. Farmer P, Connors M, and Simmons J, eds. Women, poverty, and AIDS: sex, drugs, and structural violence. Monroe, ME: Common Courage Press, 1996.
32. Fife E, Mode C. AIDS incidence and income. JAIDS 1992;5:1105-10.
33. Wallace R, Fullilove M, Fullilove R, Gould P, Wallace D. Will AIDS be contained within U.S. minority populations? Soc Sci Med 1994;39:1051-62.
34. Waldholz M. Precious pills: new AIDS treatment raises tough question of who will get it. Wall Street Journal, July 3, 1996, p. 1.
35. Centers for Disease Control and Prevention. Diphtheria outbreak--Russian Federation, 1990-1993. MMWR 1993;42:840-1,847.
36. Field M. The health crisis in the former Soviet Union: a report from the 'post-war' zone. Soc Sci Med 1995;1469-78.
37. Patz J, Epstein P, Burke T, Balbus J. Global climate change and emerging infectious diseases. JAMA 1996;275:217-33.

38. Garrett L. Public health and the mass media. Current Issues in Public Health 1995;1:147-50.
39. Centers for Disease Control and Prevention. Addressing emerging infectious disease threats: a prevention strategy for the United States. Atlanta, USA. Department of Health and Human Services, 1994.
40. Roizman B., ed. Infectious diseases in an age of change: the impact of human ecology and behavior on disease transmission. Washington, D.C.: National Academy Press, 1995.
41. Farmer P. On suffering and structural violence: a view from below. Daedalus 1996;125:261-83.
42. Krieger N, Rowley D, Herman A, Avery B, Phillips M. Racism, sexism, and social class: implications for studies of health, disease, and well-being. Am J Prev Med 1993; (Supplement) 9:82-122.
43. Navarro V. Race or class versus race and class: mortality differentials in the United States. Lancet 1990;336:1238-40.
44. Marmot M. Social differentials in health within and between populations. Daedalus 1994;123:197-216.
45. Wilson M. Travel and the emergence of infectious diseases. Emerging Infectious Diseases 1995;1:39-46.
46. Haggett P. Geographical aspects of the emergence of infectious diseases. Geogr Ann 1994;76:91-104.
47. Warner DC. Health issues at the US-Mexican border. JAMA 1991;265:242-7.
48. Small P, Moss A. Molecular epidemiology and the new tuberculosis. Infect Agents Dis 1993;2:132-8.
49. Levins R. Preparing for Uncertainty. Ecosystem Health 1995;1:47-57.
50. Latour B. The pasteurization of France. Sheridan A, Law J, trans. Cambridge, MA: Harvard University Press, 1988.
51. Global Health Situation and Projections. Geneva: World Health Organization, 1992.
52. Berkelman RL, Hughes JM. The conquest of infectious diseases: who are we kidding? Ann Int Med 1993;119:426-8.
53. Epstein PR. Pestilence and poverty--historical transitions and the great pandemics. Am J Prev Med 1992;8:263-78.
54. Packard R. White plague, black labor: tuberculosis and the political economy of health and disease in South Africa. Berkeley: University of California Press.
55. Aïach P, Carr-Hill R, Curtis S, Illsley R. Les inégalités sociales de santé en France et en Grande-Bretagne. Paris: INSERM, 1987.
56. Fassin D. Exclusion, underclass, marginalidad. Revue Française de Sociologie 1996;37:37-75.
57. Inhorn M, Brown P, eds. The anthropology of infectious diseases. New York: Gordon and Breach, 1996.
58. Krieger N, Zierler S. What explains the public's health? A call for epidemiologic theory. Epidemiology 1996;7:107-9.
59. Eisenberg L, Kleinman A. The relevance of social science to medicine. Dordrecht: Reidel, 1981.
60. Serres M. Le passage du nord-ouest. Paris: Editions de Minuit, 1980.
